# Oral Health Status and Oral Health–Related Quality of Life of First Nations and Metis Children

**DOI:** 10.1177/23800844211037992

**Published:** 2021-10-21

**Authors:** J. Lee, R.J. Schroth, M. Sturym, D. DeMaré, M. Rosteski, K. Batson, F. Chartrand, M.F. Bertone, T. Kennedy, K. Hai-Santiago, the Scaling-up Healthy Smile Happy Child Team, Pine Creek First Nation, Manitoba Metis Federation

**Affiliations:** 1 Department of Preventive Dental Science, Dr. Gerald Niznick College of Dentistry, Rady Faculty of Health Sciences, University of Manitoba, Winnipeg, MB, Canada; 2 Children’s Hospital Research Institute of Manitoba, Winnipeg, MB, Canada; 3 Department of Pediatrics and Child Health, Max Rady College of Medicine, Rady Faculty of Health Sciences, University of Manitoba, Winnipeg, MB, Canada; 4 Manitoba Metis Federation, Winnipeg, MB, Canada; 5 Pine Creek First Nation, Camperville, MB, Canada; 6 School of Dental Hygiene, Dr. Gerald Niznick College of Dentistry, Rady Faculty of Health Sciences, University of Manitoba, Winnipeg, MB, Canada; 7 Manitoba Health and Seniors Care, Government of Manitoba, Winnipeg, MB, Canada; 8 Healthy Smile Happy Child, Winnipeg, MB, Canada

**Keywords:** preschool, Indigenous peoples, dental caries, dental health survey, health status disparities, dental care for children

## Abstract

**Objectives:**

To assess the oral health status and oral health–related quality of life (OHRQoL) of young First Nations and Metis children.

**Methods:**

This cross-sectional study assessed the oral health status of Indigenous children <72 mo of age while their parents/caregivers completed a questionnaire, including the Early Childhood Oral Health Impact Scale (ECOHIS), to assess OHRQoL. Analysis included descriptive statistics, bivariate analyses, and multiple regression. A P value ≤0.05 was considered significant.

**Results:**

Overall, 146 children were recruited with a mean age of 40.1 ± 21.2 (SD) months, and 49% were male. Among First Nations children, 65.4% had early childhood caries (ECC) as compared with 45.2% among Metis children (P = 0.025). However, there was no statistically significant difference in the prevalence of severe ECC (S-ECC) between First Nations and Metis children (60.6% v. 42.9%, P = 0.051). The mean decayed, missing, and filled primary teeth (dmft) score was 4.9 ± 5.3 (range 0–20), and the mean decayed, missing, and filled surfaces (dmfs) score was 14.5 ± 20.4 (range 0–80). The total mean ECOHIS score was 4.4 ± 5.9 (range 0–25), while the mean Child Impact Section and Family Impact Section scores were 2.6 ± 4.0 (range 0–10) and 1.8 ± 2.8 (range 0–8), respectively. Multiple linear regression showed S-ECC was associated with total mean ECOHIS scores (P = 0.02). Higher total mean ECOHIS scores (which indicates poorer OHRQoL) were observed in children with ECC compared with caries-free children (5.8 v. 2.4, P = 0.0001).

**Conclusion:**

Oral health disparities such as ECC and reduced OHRQoL exist among many First Nations and Metis children in Manitoba. This is the first Canadian study exploring the OHRQoL of Indigenous children in addition to their oral health status.

**Knowledge Transfer Statement:**

This study is the first to report on the oral health–related quality of life and its relationship to early childhood caries (ECC) among young Canadian First Nations and Metis children. Metis children are just as likely to suffer from severe ECC than First Nations children. The findings of this study have informed community-based and community-developed oral health promotion and ECC prevention activities.

## Introduction

One major public health issue facing young Indigenous children in Canada is early childhood caries (ECC), which is defined as the presence of tooth decay in primary teeth in children <72 mo of age (Harrison et al. 2010). Few studies suggested that the prevalence of ECC varies from population to population, with disadvantaged children, particularly those in low-socioeconomic groups, being the most vulnerable (Dye et al. 2007; Kawashita et al. 2011). In Canada, Indigenous (First Nations, Metis, and Inuit) children are considered one group that experience significant oral health problems (Schroth and Moffatt 2005; Schroth et al. 2008; Schroth et al. 2009; Findlay and Janz 2012; Schroth et al. 2015; Pierce et al. 2019). Based on data from Health Canada on the prevalence of ECC for First Nations and Inuit children, it was reported that up to 85% of Inuit and 86% of First Nations children were affected (Health Canada 2011; The First Nations Information Governance Centre 2012). Some key determinants of ECC that have been identified among children included poverty, limited access to dental care, the absence of fluoridated water, limited access to affordable nutritious foods (Power 2008; Willows et al. 2009; Willows et al. 2011), and low education and oral health literacy (Schroth et al. 2009). Many of these risk factors are due to the historic and continuing effects of colonization and residential schools (Pascoe and Smart Richman 2009).

Many Canadian First Nations and other Indigenous children develop ECC and are disproportionally affected by severe forms of ECC known as S-ECC, which are an ongoing concern (Schroth and Morey 2007; Schroth et al. 2009; American Academy of Pediatrics, Committee on Native American Child Health, Canadian Paediatric Society, First Nations, Inuit and Métis Committee 2011; Irvine et al. 2011). For many children with S-ECC, dental surgery under general anesthesia (GA) is the only option (Schroth and Smith 2007; Canadian Institute for Health Information 2013; Schroth et al. 2016). Unfortunately, approaches that have demonstrated effectiveness in preventing ECC in lower-risk populations have not resulted in significant improvements for Indigenous children (American Dental Association 2010; American Dental Association Committee on Access, Prevention and Interprofessional Relations 2012; Braun et al. 2016). There is currently no published evidence on the prevalence of ECC in Metis children, and the oral health–related quality of life (OHRQoL) among young First Nations and Metis children in Canada is also unknown.

Psychological, social impacts of oral disease, and physical effects can all be evaluated through measurement of patient- or family-reported OHRQoL (Allen 2003; Jokovic et al. 2004; Bennadi and Reddy 2013). There are 4 domains that are assessed using the current OHRQoL model: 1) experience of dental pain/discomfort, 2) functional factors (ability to drink, chew, swallow, or speak), 3) psychological factors, and 4) social factors (comfort level when interacting with others) (Inglehart and Bagramian 2002).

The purpose of this research was to determine the burden of caries of young First Nations and Metis children in Manitoba and their associated OHRQoL. This study will help to identify the caries burden and OHRQoL of First Nations and Metis children in communities participating in an oral health promotion intervention, the Scaling up the Healthy Smile Happy Child (HSHC) initiative. This will inform our community-based and community-developed early childhood oral health (ECOH) and ECC prevention activities. It also establishes baseline findings that will be compared with assessments at the end of the study to determine the overall effectiveness of the Scaling up the HSHC initiative ECOH and ECC prevention activities.

## Methods

A cross-sectional study design was undertaken to study the oral health and associated OHRQoL in young Indigenous children in 4 Manitoba communities as part of a community-based participatory research project, the Scaling up the HSHC Initiative. This project adhered to the ethical principles of First Nations Ownership, Control, Access, and Possession and Metis Ownership, Control, Access, and Stewardship. First Nations Health and Social Secretariat of Manitoba and the Manitoba Metis Federation were partners on this study, and ethics approval was granted by the University of Manitoba’s Health Research Ethics Board.

### Data Collection

The population participating in this project was recruited from October 2018 to December 2019 and consisted of First Nations and Metis children <72 mo (6 y) of age with their primary caregiver(s). Participating communities included Winnipeg, Pine Creek First Nation (total size: 328), and 2 Metis communities located in the Northwest Metis Federation Region (referred to as Metis community 1 [total size: 487] and Metis community 2 [total size: 349]). All interested and willing parent(s) or caregiver(s) with a child <72 mo of age in each of the communities were eligible to participate in the study. The questionnaires were used to establish baseline information on knowledge, attitudes, and behaviors relating to ECOH along with OHRQoL. Overall, a total of 146 participants were recruited: Winnipeg (69), Pine Creek First Nation (18), Metis community 1 (23), and Metis community 2 (36).

Written informed consent was obtained and baseline questionnaires were completed by the parents or primary caregivers prior to completing the questionnaire and the child undergoing dental examination. The Early Childhood Oral Health Impact Scale (ECOHIS; Pahel et al. 2007) was used to assess children’s OHRQoL at the start of the study and later to assess changes in children’s OHRQoL from baseline to the end of the study. ECOHIS is a short, validated tool for use with parents and caregivers of children <72 mo of age (Pahel et al. 2007). It consists of 13 questions grouped into 2 sections: 1) Child Impact Section (CIS) and 2) Family Impact Section (FIS) (Pahel et al. 2007; Jankauskiene et al. 2014; Grant et al. 2019). Each question from the ECOHIS was scored on a scale from 0 to 4 (0 = equals never, 1 = hardly ever, 2 = occasionally, 3 = often, 4 = very often). A total ECOHIS score for each child was then calculated by summing up the responses with a maximum total score of 52 (Pahel et al. 2007; Jankauskiene et al. 2014; Grant et al. 2019). Higher scores indicate a greater negative impact on OHRQoL.

Children underwent a dental examination by trained and calibrated dentists most often in the knee-to-knee position at local community centers. Kappa statistics to determine agreement between examiners were calculated for ECC; S-ECC; decayed, missing, and filled primary teeth (dmft); and decayed, missing, and filled surfaces (dmfs) for 21 (14%) children. All eligible children and parents/caregivers who attended our community organized research events were invited to participate. Therefore, the population was sampled through convenience sampling methods as the participants were selected based on the availability and their willingness to take part in the study.

ECC and S-ECC were defined based on recognized and established case definitions formed by the American Academy of Pediatric Dentistry (Drury et al. 1999; American Academy of Pediatric Dentistry 2018). Overall dmft index (i.e., cumulative score of the total number of decayed, missing due to caries, and filled primary teeth) and dmfs index (i.e., cumulative score of the total number of decayed, missing due to caries, and filled primary tooth surfaces) scores were calculated for each child.

### Data Analysis

The child’s dental-screening results were combined with the parent’s or primary caregiver’s responses to the questionnaire. This information was then entered electronically into a REDCAP database and was analyzed using NCSS and SPSS. Descriptive statistics, bivariate analyses (chi-square analysis, *t*-test analysis, and analysis of variance) and both linear and multiple regression were performed. The main outcome variables included ECC, S-ECC, the dmft and dmfs scores, and ECOHIS scores. Covariates included the child’s sex, age, Indigenous status (i.e., First Nations or Metis), the community of residence, parental education level, insurance status, dental visit history, and S-ECC status. The independent variables considered for the regression models were deemed to be exploratory. A value of *P* ≤ 0.05 was considered statistically significant.

## Results

A total of 146 children were recruited with a mean age 40.1 ± 21.1 months, with 49% male. Most children were First Nations (71.2%) and resided in rural communities (52.7%). Most parents/primary caregivers were mothers (86.3%) and had completed high school or beyond (56.8%). Additional characteristics of participants appear in Table 1.

**Table 1.** Characteristics of Children and Their Parents or Caregivers.

Child Characteristics (*N* = 146)	*n* (%) or Mean ± SD
Mean age (mo)	40.1 ± 21.1
Sex	
Male	72 (49.3)
Female	74 (50.7)
Indigenous status	
First Nation	104 (71.2)
Metis	42 (28.8)
Place of residence	
Urban	69 (47.3)
Rural	77 (52.7)
Parental/Caregiver Characteristics (*N* = 146)	*n* (%) or Mean ± SD
Relationship to child	
Mother	126 (86.3)
Father	14 (9.6)
Grandparent	5 (3.4)
Other	1 (0.7)
Education level of caregiver (*n* = 139)	
<High school	59 (42.5)
≥High school	79 (56.8)
Declined to answer	1 (0.7)
Dental status and history (*N* = 146)	
Mean ± SD dmft^a^ score	4.9 ± 5.3
Mean ± SD dmfs^b^ score	14.5 ± 20.4
Prevalence of ECC	
Yes	87 (59.6)
No	59 (40.4)
Prevalence of S-ECC	
Yes	81 (55.5)
No	65 (44.5)

ECC, early childhood caries; S-ECC, severe ECC.

a Decayed, missing, and filled primary teeth.

b Decayed, missing, and filled surfaces.

Table 2 presents the prevalence of ECC and S-ECC by community. Agreement was calculated for 21 children, who were screened by the calibrated dental examiners. The kappa for both ECC and S-ECC was 100%. The Bland-Altman analysis was used to determine the agreement for dmft and dmfs with the correlation coefficient being 0.96 to 0.99 and 0.97 to 1.0, respectively. When the prevalences of ECC and S-ECC were compared between those living in urban and rural communities, there was no difference in prevalence of ECC in children from either type of community (*P* = 0.084). However, there was a significant difference in the prevalence of S-ECC between children living in a rural community compared with those from an urban center (*P* = 0.0057). Those from rural areas had a higher prevalence than urban-dwelling children. The prevalence of ECC and S-ECC were also compared between all 4 communities. Children from the 2 Metis communities were found to have the highest prevalence of ECC and S-ECC, whereas Winnipeg had the lowest prevalence of both ECC and S-ECC (Table 2). Overall, when the communities were compared with each other, the prevalence of ECC was not found to be statistically different (*P* = 0.34). However, the prevalence of S-ECC was found to significantly differ between communities, specifically with Winnipeg having a lower prevalence compared with the 3 rural communities (*P* = 0.0046).

**Table 2.** Prevalence by Community and Indigenous Group and Dental Caries Indices.

	ECC, *n* (%)		*P* Value	S-ECC, *n* (%)		*P* Value	dmft (Mean ± SD)	*P* Value	dmfs (Mean ± SD)	*P* Value
Variable	Yes	No		Yes	No					
Place of residence										
Urban (*n* = 69)	36 (52.2)	33 (47.8)	0.084	30 (43.5)	39 (56.5)	0.0057	3.6 ± 4.7	0.004	9.8 ± 17.8	0.008
Rural (*n* = 77)	51 (66.2)	26 (33.8)		51 (66.2)	26 (33.8)		6.1 ± 5.6		18.7 ± 21.8	
Communities										
Pine Creek First Nation (*n* = 18)	11 (61.1)	7 (38.9)	0.34	11 (61.1)	7 (38.9)	0.046	6.7 ± 6.4	<0.03	21.6 ± 24.4	0.04
Metis community 1 (*n* = 23)	16 (69.6)	7 (30.4)		15 (65.2)	8 (34.8)		5.5 ± 5.7		14.1 ± 19.5	
Metis community 2 (*n* = 36)	24 (66.7)	12 (33.3)		25 (69.4)	11 (30.6)		6.3 ± 5.1		20.2 ± 21.9	
Winnipeg (*n* = 69)	36 (52.2)	33 (47.8)		30 (43.5)	39 (56.5)		3.6 ± 4.7		9.8 ± 17.8	
Indigenous groups										
First Nation (*n* = 104)	68 (65.4)	36 (34.6)	0.025	63 (60.6)	41 (39.4)	0.051	5.4 ± 5.5	0.083	15.8 ± 21.2	0.221
Metis (*n* = 42)	19 (45.2)	23 (54.8)		18 (42.9)	24 (57.1)		3.7 ± 4.8		11.2 ± 18.2	

dmfs, decayed, missing, and filled surfaces; dmft, decayed, missing, and filled primary teeth; ECC, early childhood caries; S-ECC, severe ECC.

The associations between ECC and S-ECC prevalence and type of Indigenous groups were also assessed (Table 2). Overall, 65.4% of First Nations children had ECC compared with 45.2% among Metis children (*P* = 0.025). However, there was no statistically significant difference in the prevalence of S-ECC between First Nations and Metis children (60.6% vs 42.9%, *P* = 0.051).

Dental caries indices scores were also compared by place of residence, community, and Indigenous groups (Table 2). When dental caries indices were compared between urban- and rural-dwelling children, both the mean dmft and dmfs scores were found to be significantly higher for those from rural communities compared with children living in an urban community (*P* < 0.004 and *P* < 0.008, respectively). This analysis was then further assessed by comparing the mean dmft and dmfs scores by the individual communities (Table 2). Analysis of variance (ANOVA) showed that there were significant differences in the mean dmft and dmfs scores between communities (dmft: *P* < 0.03, dmfs: *P* < 0.04). Tukey’s analysis revealed that Winnipeg differed from the 3 rural Indigenous communities by having the lowest mean score (Table 2). When comparing the caries indices scores between First Nations and Metis children, (dmft 5.4 ± 5.5 v. 3.7 ± 4.8 and dmfs 15.8 ± 21.2 v. 11.2 ± 18.2), there were no significant differences in the mean dmft and dmfs scores between the 2 Indigenous groups (*P* = 0.083 and *P* = 0.22, respectively).

The overall number of children with available ECOHIS data was 144, with 2 participants omitted due to either missing information or caregivers hesitating to answer the survey questionnaires. The most notable ECOHIS responses were in the CIS (Table 3), in which 13.9% (combined “occasionally” and “often”) of children experienced mouth pain (question 1) and 11.1% had difficulty pronouncing any words (question 4) “occasionally,” “often,” or “very often.” In addition, 9.0% had difficulty eating (question 3) “occasionally,” “often,” or “very often.” In the FIS, 21.5% of parents reported feeling guilty (question 11) and 14.6% of parents were upset with their child’s dental condition (question 10) “occasionally,” “often,” or “very often.” The mean total baseline ECOHIS score was 4.4 ± 5.9 (0 to 30), while the mean CIS and FIS scores were 2.6 ± 4.0 (0 to 24) and 1.8 ± 2.8 (range = 0 to 8), respectively.

**Table 3.** Early Childhood Oral Health Impact Scale (ECOHIS) Responses in the Survey of Parents of <72 mo (*N* = 144).

Impact	Mean (±SD)	Never, *n* (%)	Hardly Ever, *n* (%)	Occasionally, *n* (%)	Often, *n* (%)	Very Often, *n* (%)	Don’t Know, *n* (%)
Child impact section (CIS)							
Child symptoms: question 1	**0.5 (0.9)**						
1. Oral/dental pain		97 (67.4)	26 (18.0)	17 (11.8)	3 (2.1)	0 (0.0)	1 (0.7)
Child functions: questions 2–5	**1.4 (2.3)**						
2. Difficulty drinking		114 (79.2)	20 (13.9)	8 (5.5)	1 (0.7)	0 (0.0)	1 (0.7)
3. Difficulty eating		115 (79.9)	14 (9.7)	9 (6.2)	4 (2.8)	0 (0.0)	2 (1.4)
4. Difficulty pronouncing words		114 (79.2)	7 (4.9)	9 (6.2)	4 (2.8)	3 (2.1)	7 (4.9)
5. Missed preschool or school		130 (90.3)	12 (8.3)	1 (0.7)	0 (0.0)	0 (0.0)	1 (0.7)
Child psychological: questions 6–7	**0.5 (1.1)**						
6. Trouble sleeping		120 (83.3)	17 (11.8)	7 (4.9)	0 (0.0)	0 (0.0)	0 (0.0)
7. Irritable or frustrated		117 (81.3)	13 (9.0)	11 (7.6)	2 (1.4)	0 (0.0)	1 (0.7)
Child self-image/social interaction: questions 8–9	**0.2 (0.9)**						
8. Avoided smiling or laughing		135 (93.7)	3 (2.1)	2 (1.4)	3 (2.1)	0 (0.0)	1 (0.7)
9. Avoided talking		138 (95.8)	3 (2.1)	1 (0.7)	2 (1.4)	0 (0.0)	0 (0.0)
Child section (questions 1–9)	**0.7 (1.0)**						
Family impact section (FIS)							
Parental distress: questions 10–11	**1.3 (2.3)**						
10. Been upset		106 (73.6)	16 (11.1)	8 (5.6)	3 (2.1)	10 (6.9)	1 (0.7)
11. Felt guilty		102 (70.8)	11 (7.6)	11 (7.6)	7 (4.9)	13 (9.0)	0 (0.0)
Family function: questions 12–13	**0.5 (1.2)**						
12. Time off work		121 (84.0)	14 (9.7)	6 (4.2)	2 (1.4)	1 (0.7)	0 (0.0)
13. Financial impact		129 (89.6)	4 (2.8)	6 (4.2)	3 (2.1)	2 (1.4)	0 (0.0)
Family section (questions 10–13)	**0.9 (1.4)**						
Total ECOHIS score (questions 1–13)	**4.4 (5.9)**						

When ECOHIS was compared by sex, place of residence, Indigenous status, child’s health rating, dental insurance, and dental visits, there was no significant difference in the CIS, FIS, or total mean ECOHIS scores (Table 4). When children’s ECOHIS scores were compared between communities and education level of caregivers, there was also no significant difference in the mean CIS and FIS scores (CIS: *P* = 0.159 and *P* = 0.100, FIS: *P* = 0.0518 and *P* = 0.067). However, the total mean ECOHIS scores were significantly different between the communities and education level of caregivers (*P* < 0.04, respectively for both). Tukey’s post hoc analysis revealed that Pine Creek First Nation and Metis community 2 differed from Winnipeg and Metis community 1. It was also revealed that the total mean ECOHIS scores differed significantly depending on the education level of the caregivers.

**Table 4.** Early Childhood Oral Health Impact Scale (ECOHIS) Total Score by Sex, Residence, Communities, Indigenous Status, Education Level of Caregivers, Rating of Child’s Health, ECC, S-ECC, Insurance, Dental Visits, and Rating of Child’s Dental Health.

Variable	Child Impact Section (CIS): Questions 1–9 (Mean ± SD)	Family Impact Section (FIS): Questions 10–13 (Mean ± SD)	Total Mean ECOHIS Score (Mean ± SD)
Sex			
Male	2.8 ± 4.0	1.9 ± 2.9	4.8 ± 5.9
Female	2.5 ± 4.2	1.6 ± 2.7	4.1 ± 5.8
*P* value	0.72	0.54	0.52
Place of residence			
Urban	2.3 ± 3.9	1.5 ± 2.5	3.7 ± 5.3
Rural	3.0 ± 4.2	2.1 ± 3.0	5.1 ± 6.3
*P* value	0.30	0.20	0.16
Communities			
Pine Creek First Nation	3.7 ± 5.2	2.6 ± 3.3	6.4 ± 7.0
Metis community 1	1.6 ± 2.7	0.9 ± 1.9	2.4 ± 4.4
Metis community 2	3.6 ± 4.4	2.6 ± 3.3	6.1 ± 6.6
Winnipeg	2.3 ± 3.9	1.5 ± 2.5	3.7 ± 5.3
*P* value	0.16	0.052	<0.04
Indigenous status			
First Nation	2.7 ± 4.0	1.9 ± 2.9	4.6 ± 5.9
Metis	2.6 ± 4.1	1.4 ± 2.4	4.0 ± 5.7
*P* value	0.92	0.38	0.63
Parent/caregiver rating of child’s health			
Very good	2.5 ± 4.3	1.6 ± 2.4	4.1 ± 5.9
Good	2.3 ± 3.1	1.7 ± 3.0	3.9 ± 5.2
Okay	4.7 ± 5.4	3.2 ± 4.2	7.7 ± 7.6
Poor	6.5 ± 2.1	3.0 ± 0.0	9.5 ± 2.1
*P* value	0.13	0.26	0.11
Education level of caregiver			
< High school	3.3 ± 4.4	2.2 ± 2.9	5.6 ± 6.2
≥High school	2.2 ± 3.5	1.4 ± 2.5	3.6 ± 5.3
*P* value	0.10	0.067	0.038
ECC status			
Yes	3.4 ± 4.4	2.4 ± 3.2	5.8 ± 6.6
No	1.5 ± 3.1	0.9 ± 1.6	2.4 ± 3.7
*P* value	<0.01	<0.001	<0.001
S-ECC status			
Yes	3.6 ± 4.5	2.6 ± 3.3	6.2 ± 6.7
No	1.4 ± 3.0	0.8 ± 1.6	2.2 ± 3.6
*P* value	<0.002	<0.0002	<0.0001
Dental insurance			
Yes	2.6 ± 4.1	1.7 ± 2.7	4.3 ± 5.7
No	3.0 ± 4.2	2.9 ± 4.3	5.9 ± 8.2
Don’t know	3.5 ± 0.7	3.5 ± 4.9	7.0 ± 5.7
*P* value	0.91	0.33	0.60
Parent/caregiver rating of dental health			
Very good	1.0 ± 2.4	0.4 ± 1.2	1.4 ± 2.6
Good	1.4 ± 2.6	0.5 ± 1.3	1.9 ± 3.2
Fair	3.6 ± 4.2	2.2 ± 2.4	5.8 ± 5.8
Poor	6.9 ± 5.8	6.4 ± 3.5	13.3 ± 6.5
Very poor	1.0 ± 0.0	7.0 ± 0.0	8.0 ± 0.0
*P* value	<0.0001	<0.0001	<0.0001
Previously visited a dentist			
Yes	3.3 ± 4.6	2.1 ± 2.9	5.3 ± 6.3
No	1.9 ± 3.1	1.4 ± 2.6	3.3 ± 5.1
Don’t know	0.0 ± 0.0	0.0 ± 0.0	0.0 ± 0.0
*P* value	0.21	0.35	0.19
Total (*N* = 144)	2.6 ± 4.0	1.8 ± 2.8	4.4 ± 5.9

ECC, early childhood caries; S-ECC, severe ECC.

Furthermore, ANOVA revealed that OHRQoL was associated with the prevalence of ECC (*P* < 0.001) and the prevalence of S-ECC (*P* < 0.0001). The results showed that the scores were significantly higher among children with ECC than among caries-free children. Likewise, children with S-ECC had higher mean CIS, FIS, and total mean ECOHIS scores (Table 4). When the total mean scores were compared with the rating of the child’s dental health, there were significant differences in the total mean CIS, FIS, and ECOHIS scores (*P* < 0.0001). Tukey’s analysis revealed that children who were rated either “poor” or “very poor” by their parents/caregivers on their dental health had significantly higher mean scores compared with those who rated their children’s dental health as very good or good (Table 4).

Multiple linear regression analysis was performed for total mean ECOHIS, CIS, and FIS scores. Covariates in the models included child’s sex, age, Indigenous status, community of residence, parental education level, insurance status, dental visit history, and S-ECC status. A total of 126 children were included in these analyses when observations were made with these predictor variables. When systematic basic predictors were analyzed with one another, only one predictor variable, S-ECC, was associated with total mean ECOHIS scores (*P* = 0.02; Table 5). However, stepwise regression analysis revealed that 2 of these covariates were significantly and independently associated with total mean ECOHIS scores, namely, children’s age (*P* = 0.04) and S-ECC (*P* < 0.001).

**Table 5.** Multiple Linear Regression for Baseline Total ECOHIS Scores (*N* = 126).

Variable	Regression Coefficient, B(i)	95% CI	*P* Value	Stepwise Coefficient	P Value^a^
Male sex (reference female)	0.97	−0.90, 2.83	0.31	—	—
Age (mo)	0.04	−0.01, 0.10	0.11	0.18	0.04
Metis (reference First Nation)	−0.04	−2.21, 2.12	0.97	—	—
Urban (reference rural)	−1.12	−3.17, 0.93	0.28	—	—
≥High school education (reference <high school)	−1.46	−3.42, 0.50	0.14	—	—
Insurance (reference no)	−1.44	−4.70, 1.82	0.38	—	—
Dental visit (reference no)	0.82	−1.49, 3.13	0.48	—	—
S-ECC (reference no)	2.45	0.38, 4.51	0.02	0.29	< 0.001

ECOHIS, Early Childhood Oral Health Impact Scale; S-ECC, severe early childhood caries.

a Not adjusted for stepwise regression (selection procedure).

## Discussion

This cross-sectional study assessed the oral health status of young First Nations and Metis children from 4 participating communities. Particularly, this investigation focused on the OHRQoL and prevalence of ECC, S-ECC, and caries rates.

To our knowledge, this is the first study to assess the oral health status of young Metis children in Canada. Of the total Metis children recruited, the prevalence of ECC and S-ECC was 45.2% and 42.9%, respectively (Table 2). Among First Nations children, 65.4% had ECC and 60.6% had S-ECC (Table 2). The prevalence of ECC and S-ECC among First Nations children was consistent with other previous studies (Harrison and Davis 1993; Peressini et al. 2004; Schroth et al. 2005; Harrison et al. 2006; Schroth et al. 2007; Lawrence et al. 2008; Lawrence et al. 2009; Harrison et al. 2012; Agnello et al. 2017). There was a significant difference in both the prevalence of S-ECC and caries rates between children living in rural communities compared with those from urban areas. Being consistent with prior studies, this result was not surprising, as the location of residence can significantly affect the convenience and likelihood of a family visiting a dental clinic for oral health care (Harrison and Davis 1993; Peressini et al. 2004; Schroth et al. 2005; Harrison et al. 2006; Schroth et al. 2007; Lawrence et al. 2008; Lawrence et al. 2009; Harrison et al. 2012; Agnello et al. 2017). Children in urban settings might have better oral health than their counterparts from rural communities, likely because of better access to care in urban areas.

This research was also one of the few studies in Canada to assess the OHRQoL in First Nations and Metis young children. This matters, as oral health is not only about dental status, such as caries status and caries scores, but also the impact caries has on childhood well-being, quality of life, and families. Along with the prevalence of ECC and S-ECC, the OHRQoL among First Nations and Metis young children was not previously known. That is why the self-rated ECOHIS provides a good indication of one’s self-health. The baseline total mean ECOHIS score in this study was 4.4 (Table 4). When an international comparison of baseline total ECOHIS scores was made, the children who participated in our study had similar OHRQoL to other children of different populations (Grant et al. 2019). A Canadian ECOHIS study from Quebec used a validated French version of ECOHIS, which recorded that the mean total ECOHIS for parents rating their child’s oral health as “relatively poor” was 10.8 (Li et al. 2008). Another study’s total mean ECOHIS score for pretreatment under GA was 6.3 (Grant et al. 2019). In our study, children with ECC and S-ECC had a mean ECOHIS score of 5.8 and 6.2 (Table 4). The variation in scores suggests that different factors such as culture and population need to be considered and can affect the scoring (Grant et al. 2019). The parents/caregiver’s lack of recognition or even being in denial of how their child’s dental problems are affecting their OHRQoL may also exist.

When examining individual ECOHIS questions (Table 3), although a significantly large number of participants scored at or near the lowest possible score of zero (floor effect) associated with ECOHIS, the highest scores were associated with CIS questions related to pain (question 1), eating (question 3), pronouncing words (question 4), and the FIS questions related to feeling upset (question 10) and feeling guilty (question 11) (Pahel et al. 2007). This is probably an indication that the child and caregiver have minor problems but may also be due to the survey instrument not being sensitive to problems that are experienced. There is no published ECOHIS score–related content on First Nations and Metis children; thus, there are no appropriate comparisons that can be made with these data. Even though there are no direct comparisons available, one study observed the OHRQoL of Canadian preschool children with S-ECC and had similar baseline findings, in which higher baseline scores were found within the domains of “symptoms” and “function” (CIS) and “parental distress” (FIS) (Grant et al. 2019). Along with similarities, differences in the ECOHIS scores can also result simply due to the parents/caregivers not being fully aware of all symptoms that their child is experiencing (Pahel et al. 2007). Parents/caregivers may also have a different perception of the child’s oral health compared with their child’s own awareness and even when compared between families.

In this study, the OHRQoL of First Nations and Metis children was found to be associated with the prevalence of ECC, S-ECC, and increasing caries rates (Table 4). Children from Winnipeg and Metis community 1 had significantly lower total mean ECOHIS scores as compared with Pine Creek First Nation and Metis community 2, indicating that there is a suboptimal OHRQoL, especially in rural communities. This result was not surprising because participants in urban regions would have easier access to dental services.

In addition, children with ECC and S-ECC had a significantly higher mean total ECOHIS, CIS, and FIS scores compared with those who were caries free (Table 4). The higher mean ECOHIS, CIS, and FIS scores translate to children having poorer OHRQoL (Pahel et al. 2007). A possible explanation for this is that children with more teeth are exposed to more factors that contribute to decay for a longer period compared with those whose teeth may have only just recently erupted.

Although one study limitation is the small sample size, and generalizability may be in question, nearly all of the eligible children from these 3 rural Indigenous communities participated, making the sample representative to these 3 rural communities and Winnipeg but not necessarily to other Indigenous communities. Other limitations include recall and response bias. Parents/caregivers may have had some difficulties recalling answers to the retrospective questions that were asked in the survey questionnaires about their child. Response bias is also possible since the parent’s/caregiver’s ratings about their child’s health and the answers to the ECOHIS questions may differ from the child’s own health ratings. The fact that this is a cross-sectional study can be a limitation itself, capturing only information at the time that the data were collected (baseline data); therefore, the data are bound by the timeline of the study, and inferences about causation cannot be made. Lastly, other data variables that have also been collected in this study (i.e., employment status and income) could have been incorporated and be assessed.

This study had several strengths. This was the first time that the prevalence of ECC in Metis children and the OHRQoL of First Nations and Metis children were examined in Canada using ECOHIS. As opposed to using a secondary data source, primary data were obtained through in-person collection from each of the participating communities. Appropriate statistical tests were used as they were chosen based on the nature of the main outcome variable and the covariates under consideration. The data were considered a reliable source for looking specifically at First Nations and Metis population groups in Manitoba, since the sample achieved was considered representative of these communities. Lastly, scientifically valid research was able to be achieved by using a validated scale that has internal consistency and reliability while respecting First Nations and Metis processes and goals of self-determination in this study. As long as the same method of data collection is used at baseline and follow-up (self-completed or interviewer led), reliable results can be obtained.

## Conclusion

Rates of ECC and S-ECC were prevalent, with 59.6% and 55.5% of Indigenous children participating in this study affected, respectively. Oral health disparities such as ECC and reduced OHRQoL were found to exist among many First Nations and Metis children from the 4 participating Manitoba communities. Children who had caries and resided in rural areas had poorer OHRQoL compared with those who were caries-free or resided in urban regions. These findings will help build on Indigenous knowledge relating to ECOH and OHRQoL.

## Author Contributions

J. Lee, R.J. Schroth, contributed to conception, design, data acquisition, analysis, and interpretation, drafted and critically revised the manuscript; M. Sturym, D. DeMaré, M. Rosteski, T. Kennedy, K. Hai-Santiago, contributed to data acquisition, critically revised the manuscript; K. Batson, F. Chartrand, contributed to data acquisition and interpretation, critically revised the manuscript; M.F. Bertone, contributed to data interpretation, critically revised the manuscript. All authors gave final approval and agree to be accountable for all aspects of the work.
